# Biosynthetic optical waveguide interface integration using biomimetic - *de novo* design ELP for optoelectronic applications

**DOI:** 10.1016/j.csbj.2025.12.009

**Published:** 2025-12-13

**Authors:** Anni Seisto, Ari Hokkanen, Pia Damlin, Robert Pylkkänen, Kirsi Kiiveri, Anna S. Borisova, Carita Kvarnström, Xu Cheng, Zhipei Sun, Nonappa, Pezhman Mohammadi

**Affiliations:** a VTT Technical Research Centre of Finland Ltd., VTT, 02044, Finland; b Department of Chemistry, University of Turku, Henrikinkatu 2, Turku 20500, Finland; c Department of Electronics and Nanoengineering, Aalto University, Espoo 02150, Finland; d Faculty of Engineering and Natural Sciences, Tampere University, P. O. Box 541, Tampere 33101, Finland

**Keywords:** Elastin-like polypeptide (ELP), Bioinspired materials engineering, Protein-based, Fabrication, Structural biotechnology, Sustainable biomaterials

## Abstract

The integration of biologically inspired materials into photonic device fabrication offers a promising route toward sustainable and biocompatible alternative to conventional in inorganic or petroleum based synthetic materials used in optoelectronic systems. In this work, we present a biosynthetic approach for waveguide fabrication utilizing a biomimetic - *de novo* designed elastin-like polypeptide (ELP) formulated into an all-water-based photoresist compatible with two-photon polymerization (2PP). The ELP was genetically engineered and recombinantly produced in microbes for enhanced molecular stability, a critical feature for withstanding both localized and bulk temperature increases that occur during high-intensity laser exposure during printing. The resulting ELP formulation supported direct writing of waveguide architecture without the need for organic solvents, harsh processing steps, or post-functionalization. This aqueous resist formulation exhibits high stability during printing and retains its structural integrity upon curing, making it a promising candidate for environmentally friendly, soft-material photonics. This work establishes a foundation for using biosynthetic polypeptides in the fabrication of functional photonic elements and demonstrates a step toward greener, protein-based optoelectronic manufacturing technologies.

## Graphical Abstract

**Figure:**
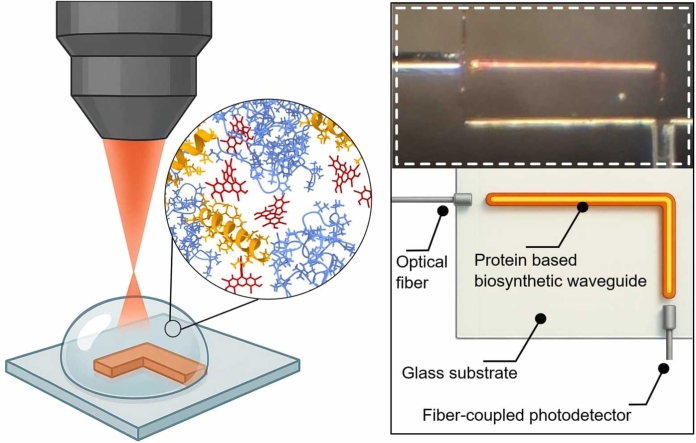

## 1 Introduction

Optical waveguides are fundamental components in modern optoelectronics systems, enabling the transmission and manipulation of light with transformative positive economic and industrial impact. [1] The global optoelectronic market reached to $46.8 billion by 2023, and it is projected to 73.83 billion by 2030, exhibiting a CAGR of 6.7 % with increasing demand for optoelectronic components in various applications such as telecommunications, consumer electronics, sensors, healthcare technology and beyond. Traditionally, waveguides have been fabricated from inorganic materials such as silicon and glass due to their low optical loss, durability, thermal and chemical stability, and scalability. However, glass-based waveguides are rigid and inflexible, and require highly sophisticated fabrication, limiting their application where flexibility and dynamically shaped devices are needed. Polymer waveguides offer tunable refractive index, facile processing and fatigue tolerant materials. [2], [3] However, they are fabricated using monomers prepared from fossil-fuel based materials, have limitations in terms of sustainability, biocompatibility, and versatility.

In this context, the convergence of biology and photonics offers a compelling route to create sustainable, functional materials for the next-generation optoelectronic devices. [4] Light-conducting elements fabricated from biologically derived or inspired biopolymers are gaining traction due to their biocompatibility, and environmental resilience. [5], [6], [7], [8], [9] Biopolymer-based optical waveguides have shown promising results for short-distance communication and sensing. [10], [11] Furthermore, they allow routes for mechanically robust, scalable optical waveguides for environmental condition monitoring and quantitative sensing with performance equal to better than capacitance-based sensors. [12], [13] Yet, the development of efficient, integrated waveguide–interfaces those that bridge soft biological materials with rigid photonic circuits remains a largely untapped area of research. [14] A key technological enabler for shaping such biosynthetic materials into precise photonic structures is two-photon polymerization (2PP), a high-resolution additive manufacturing technique that allows for direct laser writing of complex 3D micro- and nanoscale architectures. [6], [15], [16] The intense, localized heating that occurs during 2PP poses strict demands on material stability, necessitating photocrosslinkable biopolymers that can withstand thermal and photochemical stress without degrading. [17], [18]

In this work we propose a paradigm shift, the emergence of a new technological field we have labeled here as **“**Biosynthetic Photonics” (i.e., synthetic biology enabled design of photonic structures)**.** In which biosynthetic photonics refers to photonic components whose optical and mechanical properties arise from genetically encoded, *de novo* designed peptide or proteins produced via synthetic biology and biotechnological rote. This vision aims to unlock the full potential of proteins as programmable, multifunctional materials for photonic applications, offering a sustainable alternative to conventional waveguide fabrication. Among biomolecular candidates, structural proteins are especially promising. [4], [5], [6], [7] Not only do they exhibit exceptional mechanical and thermal properties, but they are also biodegradable, biocompatible, and readily produced through industrial biotechnology. [19] The inherent sequence programmability of proteins can be enabled through molecular cloning and synthetic biology that allows for atomistic control over their structure and function. Moreover, their capacity for hierarchical self-assembly offers unique advantages in building multiscale, functional architectures. [19]

Previously, we demonstrated the use of accelerated physics-informed generative workflows by combining deep neural network, and multiscale computational modeling to systematically explore the vast landscape of protein sequences to engineer new class of hybrid biomimetic - *de novo* designed elastin-like polypeptides (ELPs) and produce them using industrial biotechnology processes for diverse medical and industrial applications. [20], [21] This new class of ELP was specifically engineered for enhanced molecular stability, a critical feature for withstanding both localized and bulk temperature increases that occur during high-intensity laser exposure in two-photon printing. One protein variant named UnsELP-AI.PH45 showed potential due to thermal resilience. We hypothesized that it remains structurally intact under the intense, localized heating conditions generated by the focused laser beam, avoiding molecular degradation throughout the fabrication process. We successfully showed that ELP photocrosslinkable design and stability under laser exposure enable the direct writing of defined photonic elements using 2PP. To assess the performance and viability of the protein based biosynthetic waveguides, we undertook a comprehensive multimodal characterization post-fabrication. We investigated the structural, mechanical, chemical and optical properties of printed structures using various techniques.

This work represents a significant step toward the realization of biosynthetic photonic systems, offering a sustainable and programmable alternative to conventional photonic materials. Ultimately, our platform sets the stage for a new generation of bio-integrated optoelectronic devices spanning applications space in future.

## 2 Results and discussion

### 2.1 Protein based photoresist

**Fig. 1:**
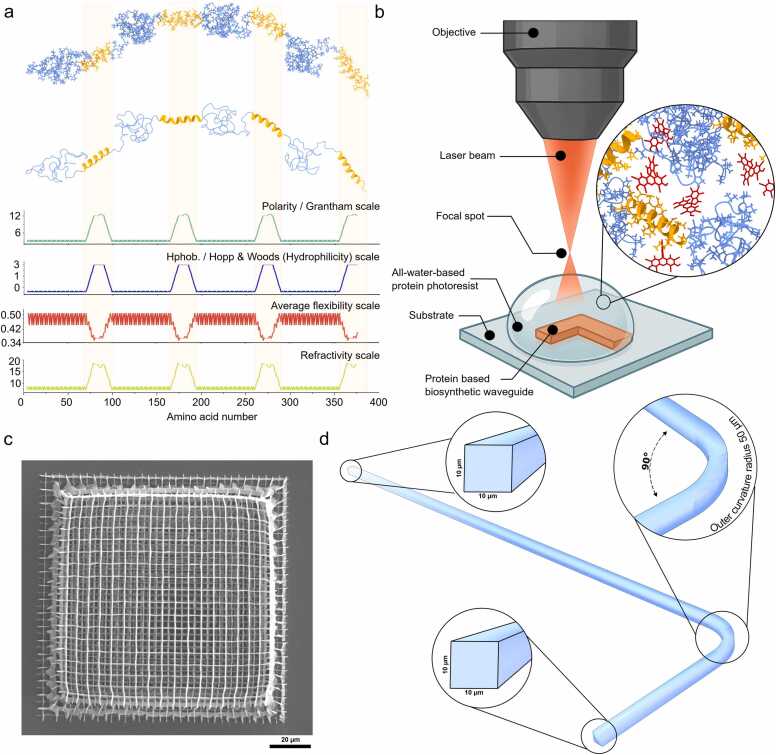
Protein-based photoresist design and two-photon 3D microfabrication of biosynthetic waveguides. a) Structural and physicochemical characterization of our biomimetic – de novo designed ELP used as the photoresist material. The top panels show a schematic representation of the protein side chain and its corresponding secondary structure, highlighting α-helical segments (orange) and disordered regions (blue). Below, quantitative plots display residue-wise properties including polarity (Grantham scale), hydrophilicity (Hopp & Woods scale), average flexibility, and refractive index potential. b) Schematic of the two-photon polymerization (2PP) setup used to fabricate biosynthetic waveguides. A tightly focused near-infrared laser beam is directed through an objective lens to induce local crosslinking within an all-aqueous protein–Rose Bengal photoresist droplet deposited on a glass substrate. The inset shows a molecular view of the protein–dye matrix under photoactivation. c) scanning electron microscopy (SEM) image of a woodpile structure printed using the optimized protein–Rose Bengal formulation and printing parameters, demonstrating sub-micron resolution. d) Microscale architecture of a printed protein waveguide. A representative L-shaped geometry is shown with insets highlighting the cross-sectional dimensions (10 µm × 10 µm) and the curvature radius (∼50 µm) of the waveguide bend.

Inspired by the hierarchical organization of natural elastins, our ELP design incorporates periodic machine learning assisted *de novo* designed α-helical domains into a naturally occurring disordered elastin-like matrix. To understand the design logic, its residue-level physicochemical properties were mapped alongside predicted secondary-structure domains (Fig.1a). The design consists of four identical 26-residue α-helical domains (orange) that recur approximately every 100 residues and are embedded within intrinsically disordered regions (blue) formed from canonical VPGVG elastin motifs. The α-helical domains were designed for improved molecular stability with added advantage by serving as rigid structural motifs, offering both mechanical tunability which may also increases in optical density that could support waveguiding photonic performance. Their positioning aligns precisely with periodic peaks in polarity, hydrophilicity, and refractivity, but contrasts with minima in average backbone flexibility, as quantified from sequence-based bioinformatic scales. These periodic physicochemical contrasts highlight a modular, bioinspired architecture in which ordered domains act as optically refractive and crosslinkable nodes, while the flanking disordered coils enable entropic elasticity and solubility. Each helix-forming segment includes tyrosine and histidine residues (YFHH), selected to enable efficient photocatalyst-mediated photocrosslinking. These residues act as reaction centers for laser-induced photopolymerization, making ELP particularly well-suited as a biosynthetic photoresist. The sequence also includes multiple lysine (K) and aspartic acid (D) residues for potential electrostatic tuning of formulation pH and ionic strength. Together, these features represent a programmable protein design strategy in which optical, mechanical, and chemical functionality are encoded into the primary sequence, suitable for biosynthetic photonic architectures built entirely from engineered peptides.

### 2.2 Protein-based photoresist design for 3D microfabrication

To render ELP photocurable we selected Rose Bengal (RB)**,** a xanthene photosensitiser that absorbs at 540–560 nm and generates singlet oxygen with near-unit quantum yield. RB has two key advantages over conventional acrylate photoinitiators: (i) it is fully water-soluble at millimolar concentrations, and (ii) its triplet lifetime (< 5 µs in water) limits oxygen-inhibition artefacts that plague radical-based systems. The ELP sequence includes four tyrosine and eight histidine residues, side chains known to form stable dityrosine and histidine-derived crosslinks under Rose Bengal mediated photo-oxidation. In addition, 6 x His tag was deliberately placed at the C-terminus of the full-length construct to enable purification via immobilized metal affinity chromatography (IMAC). These residues provide abundant reactive handles for efficient crosslinking without the need for additional chemical functionalization. Upon exposure of the RB–ELP resist to a 780 nm femtosecond laser under aqueous conditions via 2PP, the ELP maintained a dynamic yet structured conformation, enabling spatially confined crosslinking during laser exposure and allowing for precise 3D fabrication. Crosslinking occurred rapidly, producing sharply defined features with widths ranging from 200 to 500 nm (Fig. 1b and 1c). Post-print development using only Milli-Q water, without the need for organic solvents or UV curing, successfully removed unreacted protein. After ambient drying, the printed structures retained their integrity, demonstrating the formulation's suitability for high-resolution biophotonic printing.

That being said, we hypothesized that bleaching may improve the system by eliminating excess dye residues that could interfere with optical clarity or contribute to unwanted photo reactivity in the printed structures. Therefore, we removed the Rose Bengal coloration from the printed structures. Bleaching was carried out by immersing the samples in hydrogen peroxide (H₂O₂) solution. The treatment effectively decolorized the structures, resulting in a visibly transparent appearance. To further assess the impact of this treatment, we systematically compared bleached and unbleached samples with respect to their structural, mechanical, and optical properties.

### 2.3 Structural and chemical characteristics

In this work we selected L-shaped geometry as a minimal yet informative configuration to assess the light-guiding capability of the biosynthetic waveguides under realistic routing conditions, while remaining compatible with 2PP printing constraints **(**Fig. 1d). [22], [23] This layout introduces both a straight segment and a 90° bend, enabling evaluation of optical confinement and loss within a single structure. As shown in the schematic, the waveguides featured a square cross-section of approximately 10 × 10 µm and an outer curvature radius of 50 µm at the bend, parameters selected to balance fabrication resolution with manageable propagation loss. The 90° turn serves as a stress point for assessing the structural integrity and optical continuity of the printed protein waveguide, simulating routing scenarios commonly encountered in integrated photonic circuits. Furthermore, we selected this layout since it allowed efficient coupling of input and output optical fibers on orthogonal axes, facilitating alignment and reproducibility with our experimental setup. [22], [23]

**Fig. 2:**
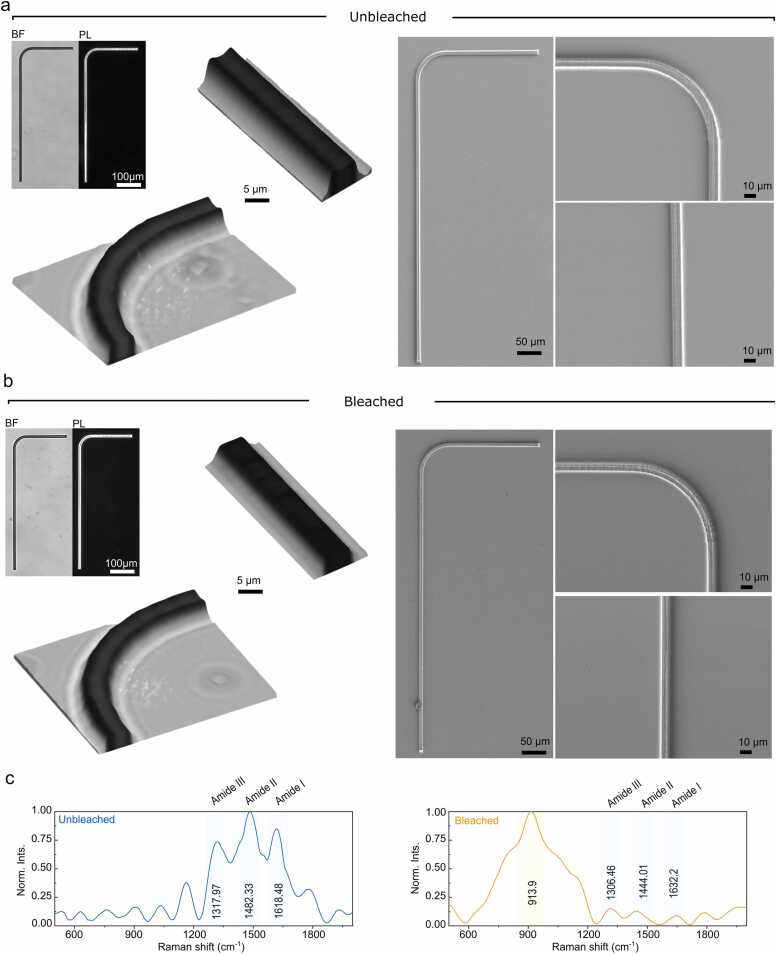
Structural and chemical characterization of printed waveguides. a) Bright-field (BF) and polarized light microscopy (PL) images (left) of unbleached waveguide. The panel also shows 3D reconstructions and scanning electron microscopy images from the waveguide cross-section, curvature and arm. b) Corresponding images for the bleached waveguides. c) Raman spectra of unbleached, and bleached waveguides normalized to their maximum intensity. Key vibrational bands corresponding to protein secondary structures are indicated, including amide III, II, and I peak, alongside bleaching-induced spectral changes.

Structural characterization of 2PP-printed L-shaped protein-based waveguides examined using bright-field (BF) microscopy, polarized light (PL) microscopy, and scanning electron microscopy (SEM) (Fig. 2a and 2b). Light microscopy confirmed that the 2PP process reproducibly yielded continuous L-shaped waveguides with smooth bends and straight segments. BF images showed well-defined edges and uniform thickness along the track, and PL microscopy revealed clearly detectable birefringence, consistent with modest molecular ordering/crystallinity established during printing (Fig. 2**a)**. SEM corroborated the LM observations, showing sharp sidewalls and an intact surface without microcracks or collapse.

After the photobleaching step, the overall morphology remained largely unchanged, suggesting that the bleaching process did not disrupt the underlying ordered structure however with subtle differences (Fig. 2**b).** The major observed differences were the refractive index, we measured this to be approximately at 1.51 ± 0.04 before bleaching and slightly lower at 1.47 ± 0.01 after bleaching. Notably, polarized-light images exhibited stronger birefringence compared with the unbleached state. We attribute this increase to a combination of (i) removal of residual chromophores that absorb/phase-shift light and damp the interference signal, thereby enhancing birefringence contrast; (ii) bleaching-induced densification/dehydration and anisotropicity; (iii) possible protein rearrangements (e.g., increased β-sheet content or additional crosslinking) that promote molecular alignment predominantly at the air water interface; and (iv) the 3D reconstructions from optical z-stacks revealed a slight reduction in thickness in the bleached samples, which could also contribute to the stronger birefringence signal by reducing light scattering and enhancing the clarity of interference patterns. Furthermore, SEM imaging indicated slightly rougher edges and surface texture after bleaching. This increased microstructural heterogeneity could locally alter light propagation and phase retardation, thereby contributing to the observed change in birefringence intensity and pattern.

To better correlate the chemical composition and structural features of the printed protein conformations, we performed Raman spectroscopy measurements on the 2PP-fabricated samples. The Raman spectra revealed distinct amide bands, amide I at 1618 cm⁻¹ (C=O stretching), amide II at 1482 cm⁻¹ (N–H bending and C–N stretching), and amide III at 1318 cm⁻¹ (C–N stretching and N–H bending), indicating that the protein backbone remains intact after 2PP (Fig. 2**c**). Despite exposure to intense laser irradiation, the spectral features confirm that the ELP retains its molecular integrity. Following bleaching with hydrogen peroxide, the amide bands remained detectable but exhibited shifts and reduced intensity - amide I to 1632 cm⁻¹ and amide III to 1306 cm⁻¹. These changes may reflect reduced hydrogen bonding, which can disrupt secondary structures. Furthermore, excessive oxidation may promote crosslinking, such as dityrosine formation from unreacted tyrosine residues during the 2PP process. This can reduce molecular mobility and enhance inter-residue interactions, leading to tighter packing and altered vibrational coupling, both of which may contribute to peak shifts and intensity loss. A new broad band at 913 cm⁻¹ appeared in the bleached sample, likely corresponding to the O–O stretching vibration of residual hydrogen peroxide, indicating incomplete removal during post-treatment. The weaker amide band intensities compared to the unbleached sample may also result from spectral interference caused by this broad peroxide signal, which can obscure sharper vibrational modes. Thus, the apparent decrease in amide band intensity may reflect both chemical modification and overlapping spectral features.

### 2.4 Mechanical properties

**Fig. 3:**
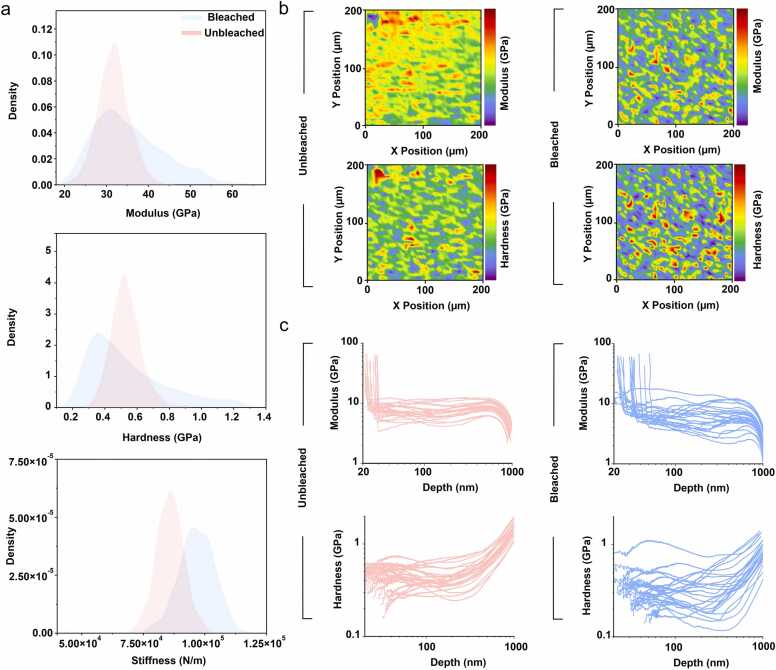
Nanoindentation test for printed geometries before and after chemical bleaching. a) Probability density plots comparing Young’s modulus, hardness, and stiffness distributions for unbleached and photobleached samples, based on nanoindentation measurements (N=1600).(N = 1600). b) Spatial maps of local mechanical properties, including modulus and hardness, for both unbleached and bleached protein films (200 μm × 200 μm). Spatial variability and mechanical contrast are color-coded for each blot. c) Depth profiles of modulus and hardness extracted from individual nanoindentation traces, shown for both unbleached (left, red) and bleached (right, blue) samples (N = 25).

To assess the mechanical integrity of the printed geometries, we performed nanoindentation to gain insight into the underlying structure–property relationships of the protein network. Measurements were carried out before and after hydrogen peroxide bleaching. Nanoindentation was selected as the primary method due to its unique capability to probe local stiffness and hardness at the nanoscale. Over 1600 individual measurements were conducted per sample to comprehensively capture mechanical variability and statistically significant differences (Fig. 3**a**). The results revealed clear distinctions in the mechanical response between bleached and unbleached protein samples, particularly in the distributions of reduced modulus and hardness. Bleached samples exhibit a broader and slightly asymmetric modulus profile, with a longer tail extending toward higher values peaking around ∼30 GPa and extending up to ∼65 GPa. In contrast, unbleached samples display a more narrowly distributed and symmetric peak, also centered around ∼30 GPa, indicating a more homogeneous material (Fig. 3**a**).

This trend is mirrored in the distribution of hardness, though with a key difference: while bleached samples show a broader spread, their average hardness is slightly lower, centered around ∼0.4 GPa, compared to ∼0.6 GPa in the unbleached group (Fig. 3**a**). Modulus more specifically the reduced elastic modulus (Er) in nanoindentation reflects the material’s resistance to elastic deformation under load. It quantifies how much the material deforms reversibly when a force is applied; a higher modulus implies a stiffer elastic response. In parallel, hardness quantifies the material’s resistance to permanent (plastic) deformation, i.e., how well the material withstands surface indentation without yielding or damage. These two parameters are intrinsic properties, derived from the slope and depth of the load–displacement curve during indentation.

Our findings suggest that while the bleached material becomes stiffer in terms of elastic response, its resistance to permanent plastic deformation is somewhat diminished. The increase in modulus following hydrogen peroxide treatment likely results from localized network stiffening, potentially due to oxidative crosslinking or densification of the protein structure after the removal of Rose Bengal. The broader modulus distribution further points to mechanical heterogeneity, possibly caused by spatial variation in bleaching efficiency or the initial distribution of Rose Bengal in the matrix (Fig. 3**b**). In contrast, the reduction in average hardness may indicate that the bleaching process introduces brittle characteristics, reducing the material's capacity to absorb plastic deformation before failure. It is also possible that partial oxidative degradation of side chains, or subtle disruption in molecular packing, could soften the material under plastic loads while still increasing the overall elastic stiffness.

The kernel density estimation of stiffness values further substantiates the hypothesis of structural reinforcement upon bleaching (Fig. 3**a**). Bleached samples not only exhibit a higher average stiffness (∼95,000–105,000 N/m) but also show a broad tail toward higher stiffness values, around 120,000 N/m, indicative of local regions with enhanced crosslinking or structural compaction. In contrast, unbleached samples display a narrower, symmetric distribution centered around ∼85,000 N/m, pointing to a more uniform network (Fig. 3**a**). Stiffness, unlike modulus or hardness, is an extrinsic parameter measured directly as the slope of the initial unloading curve. It reflects the force required to achieve a given displacement in the context of both the sample and the probe. In homogeneous materials, stiffness correlates with modulus; however, in patterned or heterogeneous materials such as these protein structures, stiffness can reveal local mechanical variations and mesoscale effects, including differences in network density, crosslinking heterogeneity, and the presence of residual solvent or embedded unreacted photoresist.

To complement the earlier observations, we then performed continuous stiffness measurements (CSM) as a function of indentation depth for both bleached and unbleached protein structures (Fig. 3**c**). These measurements provide detailed insight into how elastic and plastic properties evolve through the vertical profile of the printed material. For both samples a gradual decrease for modulus observed as a function of indentation depth. The unbleached sample displayed a more consistent gradual decline stabilizing at around ∼10 GPa between depth of 100–700 nm. The individual curves show relatively low variability, suggesting that the mechanical properties are homogeneous through depth. In contrast, bleached samples exhibited a broader range of behaviors. Furthermore, hardness exhibited similar trends of depth-dependent decay.

In summary, nanoindentation analysis demonstrates the dual impact of hydrogen peroxide treatment. Bleaching alters the mechanical landscape in a non-uniform manner, enhancing elastic stiffness in select regions while slightly compromising the material’s average plastic resilience. It not only increases the average mechanical strength of 2PP-printed protein structures but also leads to the emergence of spatially heterogeneous domains with higher mechanical properties. Hydrogen peroxide likely contributes to secondary crosslinking or oxidative stabilization of the protein matrix, resulting in tighter packing or stronger inter-residue interactions, such dityrosine bonds of unreacted tyrosine residues. In contrast, when Rose Bengal is retained in the printed structures, it may act as a mechanical plasticizer, disrupting network formation or introducing local strain fields that reduce effective modulus and hardness. Upon bleaching, its removal allows the protein matrix to relax and reorganize, forming tighter, more cohesive domains that better resist both elastic and plastic deformation. Additionally, hydrogen peroxide is known to facilitate oxidative crosslinking reactions in protein systems, which could further increase overall network stiffness and rigidity.

### 2.5 Optical measurements

**Fig. 4:**
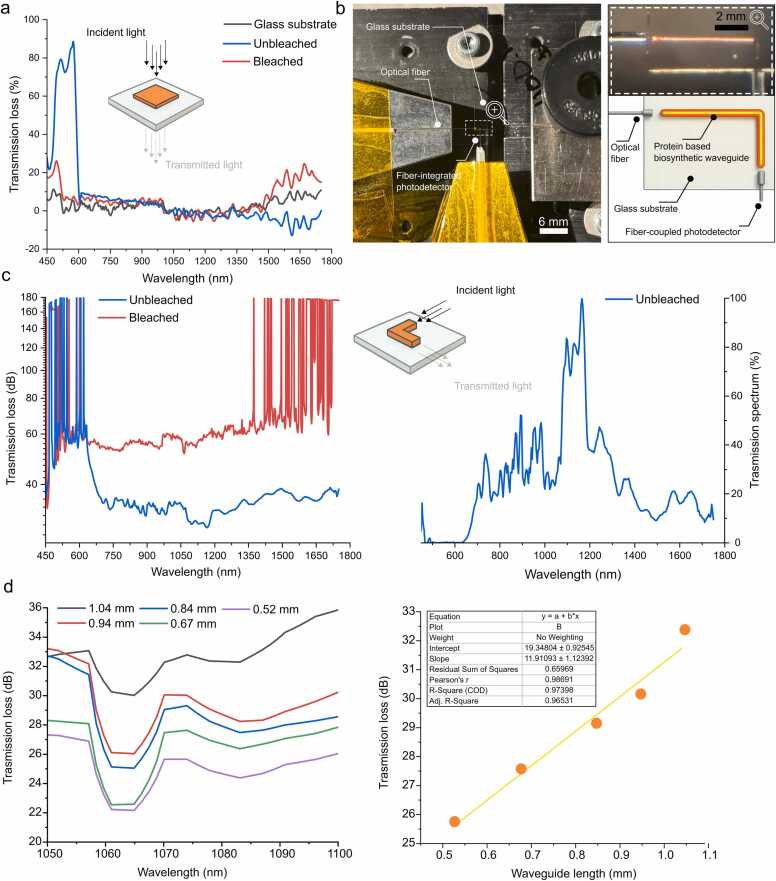
Optical characterization of biosynthetic protein-based photonic structures. a) Transmission loss spectra of protein square films measured across the 450–1750 nm range, comparing unbleached (blue) and bleached (red) samples with a glass substrate control (black). b) Optical micrograph and schematic of an L-shaped biosynthetic waveguide fabricated using two-photon polymerization on a glass substrate. The waveguide was illuminated from the left via a single-mode optical fiber, with light guided through a 90° bend and collected at the output using a fiber coupled photodetector. c) Left: Spectral transmission loss of unbleached and bleached L-shaped waveguides measured from 450 to 1800 nm. Right: Absolute transmission spectrums showing detectable propagation in the near infrared. d) Left: representative length-dependent transmission loss spectra measured in the 1050–1100 nm range for unbleached waveguides of varying lengths (0.52–1.04 mm), showing increasing loss with length. Right: Linear fit of transmission loss vs. waveguide length reveals a propagation loss of ∼11.9 dB/mm (R² = 0.97) at 1070 nm, indicating consistent attenuation with increasing waveguide length. All measurements were performed as triplicates, mean and STD are shown (note: STD may not be visibly distinguishable due to small measurement error).

To evaluate the intrinsic optical properties of the printed biosynthetic material and to establish a reference for more complex photonic elements, we first investigated the light transmission characteristics of simple protein square geometries. These structures offered a straightforward and optically uniform platform, facilitating direct comparison of material modifications—specifically, the impact of the bleaching process—under controlled measurement conditions. Fig. 4**a** presents the transmission loss spectra for protein square films in the 450–1750 nm wavelength range, comparing unbleached (blue curve) and bleached (red curve) samples with a bare glass substrate (black curve) as a baseline. The unbleached sample shows pronounced loss in the visible region, with a sharp attenuation peak exceeding 80 % around 550–600 nm, attributable to residual Rose Bengal dye used in the photopolymerization process. In contrast, the bleached sample demonstrates significantly reduced loss in this region, confirming effective dye removal through hydrogen peroxide treatment. Across the near-infrared range (700–1750 nm), both samples exhibit broadly similar trends, but the bleached sample consistently shows higher attenuation, especially above 1400 nm where unbleached samples demonstrate decreasing loss. These findings confirm that bleaching improves transparency across a broad spectral range, reducing absorptive and scattering losses. This baseline characterization informed subsequent measurements in structured, functional photonic elements, including the L-shaped waveguides described below.

Following the baseline characterization, we next evaluated the light-guiding performance of functional photonic L-shaped structures. Fig. 4**b** illustrates the experimental setup and physical design of the L-shaped protein-based biosynthetic waveguide, fabricated via 2PP directly on a glass substrate. This geometry was chosen to demonstrate both straight and bent light propagation pathways within a single structure while minimizing fabrication complexity and alignment challenges. The configuration involved coupling a supercontinuum light source into the waveguide via a single-mode optical fiber and collecting transmitted light at the output with a fiber coupled photodetector. The upper part of the panel shows an optical micrograph of the waveguide under illumination, where visible red-orange emission indicates successful light coupling, internal propagation, and out-coupling at the distal end. The bright internal guiding path confirms that the biosynthetic material supports confined light transmission even across a 90° bend, without significant visible leakage or scattering at the corner. The lower schematic illustrates the optical path and experimental layout, highlighting the L-shaped trajectory of the waveguide. This result validates the feasibility of using the printed ELP-based formulation to create functional waveguides that operate in the visible regime, and it serves as a precursor to more quantitative spectral transmission analysis shown in subsequent panels.

To further quantify the light-guiding performance of the biosynthetic protein waveguides, we measured the spectral transmission of the L-shaped structures across the 450–1750 nm range. Fig. 4**c** shows both the wavelength-dependent transmission loss for unbleached (blue curve) and bleached (red curve) samples and also presents their corresponding transmission spectrum. In the visible range (450–700 nm), both samples show considerable attenuation, consistent with the absorption behavior observed in the square film measurements (Fig. 4**a**). However, the unbleached waveguide demonstrates lower apparent transmission loss than the bleached one, which is likely due to differences in out-coupling efficiency, alignment artifacts, material performance particularly since the absolute transmission of the unbleached sample (right panel) remains low across the full spectrum. In the near-infrared range (700–1750 nm), the unbleached waveguide shows localized peaks in the transmission spectrum, with a broad maximum near 1100 nm. These features indicate partial spectral transparency, suggesting the material's potential utility in short-wave IR applications. However, the absolute transmission remains in the 0.01-0.1% range, emphasizing the need for further optical optimization.

To quantify propagation losses and assess the scalability of light transmission in the biosynthetic material, we performed a systematic length-dependent study on unbleached waveguides. Given that bleached samples exhibited poor light-guiding behavior, potentially due to microstructural changes during the bleaching process, this analysis focused exclusively on unbleached waveguides. Fig. 4**d** shows the transmission loss spectra for L-shaped waveguides of varying lengths (0.52–1.04 mm), measured in the 1050–1100 nm range. A clear trend of increasing attenuation with length is observed, with all curves exhibiting a local minimum around 1060–1070 nm, indicating a wavelength region of relatively lower propagation loss within the near-infrared window. The plot of average transmission loss as a function of waveguide length reveals a linear relationship with high correlation (R² = 0.97398). The slope of the linear fit corresponds to a propagation loss of approximately 11.9 dB/mm at 1070 nm, which is within the expected range for unoptimized soft material waveguides and serves as a baseline for future performance improvements. The relatively high intercept (∼19.3 dB) suggests substantial input/output coupling losses, likely due to mode mismatch between the single-mode fiber and the printed biosynthetic structure but also quality of prints which could have adverse effect on transmission. These results highlight both the potential and current limitations of the unbleached protein waveguides. While the material supports guided light propagation in the near-IR regime, the observed attenuation underscores the need for further optimization in waveguide geometry, printing quality, material formulation, and coupling strategy.

We also acknowledge that the measured propagation loss (∼11.8 dB/mm) may considered higher than in conventional polymer or silica-based waveguides. However, we argue that this value should be considered as a baseline benchmark for biosynthetic protein formulations, where attenuation is presently dominated by technical factors such as geometry, coupling mismatch, and residual dye rather than intrinsic material limitations. This provides a foundation for systematic optimization, including cladding incorporation, protein sequence tuning, and geometry refinement, which are expected to reduce losses significantly in future iterations.

We also emphasize that the present work was designed as an experimental feasibility study to establish protein-based formulations as functional photonic materials. The L-shaped geometry was chosen as a minimal, informative layout to test straight and bent guiding while maintaining experimental simplicity. Future efforts will combine such experimental platforms with optical simulations (e.g., finite-difference time-domain (FDTD), eigenmode analysis) and expanded device designs, including straight channels, Y-splitters, and resonators, to enable a more comprehensive assessment of photonic performance in integrated contexts.

The large intercept (∼19.3 dB) observed in the loss–length plot primarily reflects coupling inefficiencies (mode mismatch, Fresnel reflections) and interface-related effects such as facet roughness (Fig. 4d). These losses are extrinsic to the material and can be significantly reduced in future work through cladding incorporation, facet optimization, and improved coupling strategies.”

## 3 Material and methods

### 3.1 Biomimetic – *de novo* design of elastin like polypeptide (ELP)

The AIMS protocol with deep learning-based framework employed for *de novo* design of α-helical protein sequences described in our earlier work. [20], [21] Briefly, the protocol consists of three key components: AIMS-GATHER, AIMS-GENERATE, and AIMS-PROT. AIMS-GATHER performs initial data mining to identify structural or sequence-based homologs aligned with user-defined design objectives. When homologs are unavailable, DSSP is used to estimate hydrogen bonds and assign secondary structure, followed by calculation of Ψ and Φ dihedral angles. The resulting annotated dataset serves as input for the deep learning models. AIMS-GENERATE is a convolutional neural network (CNN) trained on a dataset of 95,000 protein sequences annotated with physicochemical properties. Given a target secondary structure and desired property profile, AIMS-GENERATE produces novel candidate sequences. These were filtered based on computed properties and similar thresholds to ensure novelty and compliance with design requirements. AIMS-PROT, also CNN-based, predicts the secondary structure and Ψ/Φ angles of generated sequences. It functions as a decoder in the architecture, validating that the predicted structures align with the target conformation. Sequences failing to meet a predefined accuracy threshold (e.g., 0.9) were discarded. Finally, filtered sequences subjected to atomistic molecular dynamics simulations (energy minimization and relaxation) to assess structural stability. A final similarity screen against databases such as UniProt ensures novelty of the selected candidates. A full-length sequence was engineered by incorporating fifteen times repeat of commonly used unstructured ELP sequence (VPGVG)_15_ followed by a single AIMS-PROT predicted helices (AI.PH) with identification number 45. [20], [21] The tandem repeat of the (VPGVG)_15_ and the AI-predicted helix name was then repeated four times to create a full-length protein sequence named UnsELP-AI.PH45.

### 3.2 Molecular cloning, protein expression and purification

The synthetic coding fragment was codon optimized cloned in frame with the C-terminal 6 ×His-tag for seamless golden gate cloning in pEt-28a (+) (kanR) protein expression vector for expression in *E. coli* as described previously. [24], [25], [26], [27], [28], [29], [30] 10-beta competent *E. coli* strain was used for cloning purposes and BL21 T7 express™ for expression. Luria-Bertani (LB)-agar plates and LB-medium were used with kanamycin (50 μg/mL) and ampicillin (100 μg/mL) when appropriate during cloning and expression.

For protein expression, single colonies from overnight LB agar plates were used to inoculate 5 mL of LB medium containing 50 μg/mL kanamycin. After incubation at 37 °C and 250 rpm for 6–7 h, the starter culture was transferred into 500 mL of fresh LB medium in a 2 L Erlenmeyer flask. Cultivation continued under the same conditions until cells reached mid-log phase (OD₆₀₀ ≈ 0.4). Protein expression was then induced with 0.1 mM IPTG (Sigma-Aldrich), and the culture temperature was reduced to 18 °C. Following overnight incubation (15–20 h), cells were harvested by centrifugation (16,000 ×g, 15 min, 4 °C) and either stored at −80 °C or kept at 4 °C for immediate purification.

For purification, cell pellets from 500 mL cultures were resuspended in 5 mL of lysis buffer containing 20 mM HEPES (pH 7.5), 200 mM NaCl, 20 mM imidazole, 5 mM MgCl₂, 0.5 mg/mL lysozyme, 0.01 mg/mL DNase I, and a protease inhibitor cocktail (EDTA-free). After 45 min of incubation at 4 °C with gentle mixing, cells were lysed by sonication (Qsonica 500; 20–30 % amplitude, 3 min total, with 2-second pulse cycles) while kept on ice. Lysates were clarified by centrifugation (16,000 ×g, 60 min, 4 °C), and the supernatants were filtered through 0.2 μm membranes. The clarified lysate was loaded onto a HisTrap FF column (Cytiva) connected to an ÄKTA Pure FPLC system (GE Healthcare) operated at 4 °C. Proteins were purified via immobilized metal affinity chromatography (IMAC) using a low-imidazole binding buffer (20 mM imidazole, 200 mM NaCl, pH 7.4) and eluted using a gradient increasing to 200 mM imidazole, as defined in UNICORN 7 software. Buffer exchange was performed using Econo-Pac 10 DG desalting columns (Bio-Rad) with 50 mM Tris-HCl (pH 7.4). Final protein samples were washed and concentrated using 10 kDa MWCO Vivaspin spin concentrators. Protein concentration was quantified by absorbance at 280 nm using a DS-11 FX spectrophotometer (DeNovix), and purity was assessed by SDS-PAGE (4–20 % gradient gels; Bio-Rad). Gels were stained with Coomassie Brilliant Blue and visualized using a Bio-Rad ChemiDoc XRS system. Samples were stored at −80 °C unless used immediately.

### 3.3 Printing formulation

100 µL of printing formulations were prepared by mixing protein solution (5–6 % w/v) was mixed with 40 µL of Rose Bengal solution (20 % w/v), yielding a final protein concentration of ≈ 3.6–4.3 % (w/v) and Rose Bengal concentration of ≈ 5.7 % (w/v) in the printing droplet. The 5–6 % range reflects batch-to-batch variation in protein stock concentration, while the mixing ratio was fixed. At these concentrations the formulation remains a low-viscosity aqueous solution that can be easily spread into a thin film or droplet therefore no rheological characterization performed on the printing formulation.

### 3.4 Design of 2D model

The 2D models used for printing were designed using computer-aided design (CAD) software. The models were created using AutoCAD (Autodesk Inc., USA) or Fusion 360 (Autodesk Inc., USA) unless otherwise stated. The cube model measured 500 × 500 µm with a thickness of 5 µm The L-shaped waveguide consisted of a long arms measuring 1400 µm, 1100 µm, 800 µm or 500 µm and the short arm of 200 µm. Both long and short arms with a thickness of 10 µm. The corner of the L-shape was rounded, with an outer curvature radius of 50 µm and an inner curvature radius of 40 µm. All models were designed as a solid structure.

### 3.5 Two-photon polymerization (2PP)

Two-photon polymerization (2PP) printing was carried out using a NanoOne 250 (UpNano GmbH, Austria) 3D printing system. The models were printed onto 0.17 mm thick glass coverslips, which were cleaned with ethanol prior to use. The printing formulation was pipetted directly onto the glass surface, with 5 µL used for waveguide models and 10 µL for cube models. During printing, the infill mode was set to "fineline", and the direction was adjusted to 0–90° depending on the model and the model’s printing angle. The infill power was maintained at 65 mW, with an infill line distance and layer height of 0.7 µm, and a printing speed of 9 mm/s. Following the printing process, the samples were immersed in water for 20 min to remove any uncross linked ELPs. After the cleanup step, the samples were either dried directly at ambient conditions (unbleached) or subjected to a bleaching process. For bleaching, 300 µL of 30 % hydrogen peroxide was applied onto the printed structures, which were then exposed to LED light for 1 h and 20 min. The samples were subsequently rinsed in water for 2 min to remove residual hydrogen peroxide and then dried.

### 3.6 Nanomechanical measurement

Nanomechanical testing was performed using an iNano® nanoindenter (KLA Corporation, USA) equipped with a Berkovich diamond tip (Synton MDP, Switzerland) and an InForce 50 electromagnetic actuator. The system included modules for continuous stiffness measurement (CSM) and high-speed 2D mechanical mapping (NanoBlitz 3D). The diamond area function (DAF) of the indenter was calibrated using the Oliver and Pharr method. [31], [32] The Advanced Dynamic E&H method was used to characterize the relationship between hardness and elastic modulus. Measurements were conducted with a target load of 50 mN, an indentation strain rate of 0.200 s⁻¹ , frequency of 110 Hz, and a target displacement amplitude of 2 nm. A total of 400 data points were collected per measurement. For spatially resolved mechanical analysis, the E&H Mapping method with NanoBlitz 3D was employed. A 100 × 100 µm area was mapped using a target load of 3 mN and a data point spacing of 200 µm, generating 40 measurement points per map.

### 3.7 Raman spectroscopy

Raman measurements were performed using a Renishaw inVia confocal Raman microscope (Leica). A 633 nm laser was employed for excitation, with laser power intensities ranging from 1 % to 100 %, depending on the sample sensitivity. Raman spectra were acquired using either a 20x or 50x objective lens unless otherwise stated. The Raman-scattered light was dispersed using an 1800 lines/mm diffraction grating and detected with a Peltier-cooled charge-coupled device (CCD) detector. Spectra were collected over a range of 100–2000 cm⁻¹ to encompass characteristic vibrational modes of the protein backbone and dye residues. The spectrometer was calibrated using a silicon standard with a reference peak at 520.5 cm⁻¹ . Samples were mounted on clean glass slides and scanned without additional coatings or fixatives. Two measurement modes were used: extended scans were recorded with 10-second acquisition times and 1–3 accumulations, while static scans were performed with 1-second acquisition times and 6–12 accumulations, depending on the signal-to-noise requirements.

### 3.8 Scanning electron microscopy

Zeiss FE-SEM field emission microscope operated at 2–3 kV under variable pressure was used for SEM imaging as described previously. [25], [29], [33], [34], [35], [36], [37], [38] Prior to imaging, samples were coated with a 30 nm layer of platinum using a sputter coater to enhance conductivity and eliminate the charging effect.

### 3.9 Light microscopy

Light microscopy was performed using a Zeiss Axio Observer 7 microscope (Carl Zeiss AG, Germany), equipped with 5X, 10X, and 20X objective lenses. The microscope was used to assess the structural integrity, geometry, and surface quality of the samples after printing.

### 3.10 Optical measurement

Supercontinuum light source (NKT Photonics SuperK compact) was coupled to single mode fiber (9/125 µm core and cladding diameter) in resist and in protein square transmission measurements. Pulse frequency of the supercontinuum was 1500 Hz. Light was collimated through protein squares with Thorlabs’ Fiber-to-Fiber U-Bench (FBP-B-FC). Transmitted light was coupled to an optical spectrum analyzer (OSA) (Ando Electric Ltd., AQ-6315A) using multimode fiber (400/425 mm core/cladding diameter). OSA measured transmitted light in the 450–1750 nm wavelength range using 10 nm resolution. Transmission range and attenuation of protein waveguides were measured with the same supercontinuum light source and OSA. Now single mode fiber (9/125 µm core and cladding diameter) was coupled directly to protein waveguide and transmitted light was coupled to OSA using multimode fiber (105/125 µm core and cladding diameter). OSA measured transmitted light in the 450–1750 nm wavelength range using 10 nm resolution. Pulse frequency of the supercontinuum was 1500 Hz for unbleached protein waveguides and 1500/10000 Hz for bleached protein waveguides. Attenuation was measured with cut-back method using South Bay Technology Inc. Lapping & Polishing Machine (Model 920) to decrease the waveguide length. Griding was made with Allied High Tech Products Inc. diamond lapping films with 9 µm particles without any polishing steps. All measurements were performed as triplicates, mean and STD are shown.

### 3.11 Ellipsometry

Spectroscopic Ellipsometer SE-2000 (2F09 05) was used for the RI measurements. The system operated in Ultramicrospot mode, which provides a minimum spot size of approximately 60 µm. For data fitting and analysis, the Spectroscopic Ellipsometry Analyzer (SEA) software was employed, supporting models such as Cauchy/Lorentz for refractive index extraction.

## 4 Conclusion

In this study, we demonstrated the using of a *de novo* designed ELP as functional protein-based materials for high-resolution photonic microfabrication via 2PP. The ELP, designed through our previously established AIMS protocol, an unsupervised deep learning-based framework, integrates periodic α-helical domains within a disordered elastin backbone, yielding a protein matrix with programmable mechanical, optical, and crosslinking characteristics. This design enabled the formulation of a fully aqueous, single-component photoresist system with Rose Bengal, compatible with sub-micron 3D printing under femtosecond laser exposure. The printed biosynthetic structures exhibited structural integrity and light-guiding functionality. Structural, chemical, and mechanical analyses confirmed that the printed designs retained molecular integrity and exhibited sufficient stiffness, uniformity, and optical responsiveness for photonic applications. Optical transmission studies validated near-infrared light propagation in L-shaped geometries, establishing a functional baseline for waveguiding in protein-based materials. We confirmed near-infrared light guiding with a baseline propagation loss of ∼11.9 dB/mm. Although this loss is higher than in optimized polymeric or inorganic systems, it establishes a realistic starting point for soft-matter photonics and supports the feasibility of protein based biosynthetic materials for light-based applications. Post-processing treatments, such as hydrogen peroxide bleaching, were explored to enhance optical transparency, revealing important structure–property trade-offs that can guide future optimization. Rather than focusing solely on these process variations, this work highlights a broader advance such as the convergence of computational protein design, sustainable material chemistry, and additive manufacturing to realize a new class of biosynthetic photonic systems. Furthermore, the work demonstrate feasibility with a single designed ELP sequence. The programmability emphasized here refers to the computational design framework, which enables rational variation of protein sequence to adjust mechanical and optical properties. Systematic experimental comparison of distinct ELP variants should be subject of follow-up studies. Altogether, this work establishes a blueprint for sequence-encoded, biodegradable photonic devices at the molecular level and compatible with environmentally benign fabrication. These findings may lay the groundwork for future efforts aimed at improving print fidelity, waveguide performance, and formulation chemistry to support biodegradable, and integrable soft-photonic systems. It also demonstrates that structural proteins can be rationally designed to meet the demands of advanced fabrication methods, and that their post-fabrication behavior optical, chemical, and mechanical can be tuned. More than materials innovation, this work introduces biosynthetic photonics, a paradigm in which biological design principles and optical device engineering co-evolve.

## CRediT authorship contribution statement

**Anni Seisto:** Writing – review & editing. **Ari Hokkanen:** Writing – review & editing, Validation. **Pia Damlin:** Writing – review & editing. **Robert Pylkkänen:** Writing – review & editing. **Kirsi Kiiveri:** Writing – review & editing. **Anna S. Borisova:** Writing – review & editing. **Carita Kvarnström:** Writing – review & editing. **Xu Cheng:** Writing – review & editing. **Zhipei Sun:** Writing – review & editing. **Nonappa:** Writing – review & editing. **Pezhman Mohammadi:** Writing – review & editing.

## Authorship contribution statement

Anni Seisto: Writing – original draft, Methodology, Investigation, Formal analysis, Data curation. Pia Damlin: Investigation, Formal analysis, Data curation. Ari Hokkanen: Writing – original draft, Validation, Methodology, Investigation, Formal analysis, Data curation, Conceptualization. Zhipei Sun: Writing – original draft, Validation. Pezhman Mohammadi: Writing – original draft, Validation, Supervision, Resources, Project administration, Funding acquisition, Conceptualization.

## Authorship contribution statement

Anni Seisto: Writing – original draft, Methodology, Investigation, Formal analysis, Data curation.

Pia Damlin: Investigation, Formal analysis, Data curation.

Ari Hokkanen: Writing – original draft, Validation, Methodology, Investigation, Formal analysis, Data curation, Conceptualization.

Robert Pylkkänen: Writing – original draft, Validation.

Kirsi Kiiveri: Writing – original draft, Validation.

Anna S Borisova: Writing – original draft, Validation.

Carita Kvarnström: Writing – original draft, Validation.

Xu Cheng: Writing – original draft, Validation.

Nonappa: Writing – original draft, Validation.

Zhipei Sun: Writing – original draft, Validation.

Pezhman Mohammadi: Writing – original draft, Validation, Supervision, Resources, Project administration, Funding acquisition, Conceptualization.

## Declaration of Competing Interest

The authors declare that they have no known competing financial interests or personal relationships that could have appeared to influence the work reported in this paper.

## Data Availability

Data will be made available on request
